# The future of Parkinson’s care: a need to expand access

**DOI:** 10.3389/fneur.2025.1622283

**Published:** 2025-06-19

**Authors:** Sana Aslam, Anjail Z. Sharrief, Samantha K. Holden, Michelle Fullard, Jodi S. Holtrop, Anna M. Maw, Amy W. Amara

**Affiliations:** ^1^Department of Neurology, University of Colorado, Aurora, CO, United States; ^2^Department of Neurology, McGovern Medical School, Institute for Stroke and Cerebrovascular Disease, The University of Texas Health Science Center at Houston, Houston, TX, United States; ^3^Department of Family Medicine, School of Medicine, University of Colorado, Aurora, CO, United States; ^4^Adult and Child Center for Outcomes Research and Delivery Science (ACCORDS), University of Colorado, Aurora, CO, United States; ^5^Division of Hospital Medicine, University of Colorado, Aurora, CO, United States

**Keywords:** Parkinson’s disease, access to care, referral pathways, eConsult, movement disorders

## Abstract

Parkinson’s disease (PD) affects over one million Americans, with prevalence expected to double by 2040, creating rising challenges for healthcare systems. While neurologist-led care, particularly by movement disorder specialists (MDS), is associated with improved patient outcomes, only a small fraction of PD patients access this level of expertise. Many, various, barriers lead to delays or missed opportunities for advanced treatments such as deep brain stimulation and infusion therapies. This Perspective article issues a call to action for improving referral pathways and care coordination in PD, addressing both clinical and systems-level gaps. We propose several pragmatic strategies, including the development of standardized referral criteria supported by clinical decision tools, expanded use of telemedicine and eConsult platforms, and enhanced provider and patient education to promote timely and appropriate access to specialty care. As early diagnostic technologies become more available, the need for structured referral pathways will become even more critical.

## Introduction

Parkinson’s disease (PD) affects approximately 1 million Americans, with prevalence projected to double by 2040, creating an escalating burden on healthcare systems. Globally, the number of individuals with PD increased by 118% between 1990 and 2015, and this figure is expected to nearly triple in the next 15 years (1, 2). Several studies highlight the critical role of neurologist-led care in improving outcomes in PD (3, 4). Patients managed by neurologists are 21% less likely to be discharged to a skilled nursing facility and 14% less likely to experience a hip fracture, even after accounting for demographic, clinical, and economic factors (4). Evaluations by movement disorders specialists (MDS) in particular facilitate screening for advanced therapies including deep brain stimulation, infusion therapies, and lesional therapies, which have been shown to be cost-effective in long-term studies, yet are significantly underutilized (5). However, access to subspecialty care is not always available, offered, or feasible.

The disparities in specialist access are striking. In some states, the median distance to the nearest movement disorder center exceeds 100 miles, creating significant barriers for many patients (6). Data from Medicare beneficiaries show that only 9% of PD patients receive care from an MDS, while 50% are treated by general neurologists, and 29% rely solely on primary care providers (PCPs) (7). These differences likely result from a combination of logistical, structural, and provider-level factors, including geographic distance, socioeconomic constraints, systemic fragmentation, and clinician perceptions or knowledge gaps, that together limit equitable referral to specialty care (7–14).

Addressing these barriers requires overcoming the issues that create them by developing innovative, scalable solutions such as streamlining communication with specialists, reducing delays, and improving care coordination, especially for high-risk patients who face the greatest roadblocks. Without a structured, standardized referral system, many PD patients will continue to experience fragmented care, resulting in poorer outcomes and increased healthcare costs. Efforts to standardize referral pathways, expand access through telemedicine, and address socioeconomic barriers can help reduce gaps in care. Educating both providers and patients may further support timely and appropriate access to PD specialists.

## Primary care physicians (PCP) and neurologists

For many patients with early PD, the first point of contact is a PCP. However, even among MDS, diagnostic accuracy in early PD is imperfect, with studies suggesting misdiagnosis rates of up to 20% in early-stage cases (15). If subspecialists, who have extensive training in PD, face challenges in early diagnosis, the difficulty is even greater for PCPs, who must manage a broad range of conditions in time-constrained clinical settings.

Even when a patient is referred to a general neurologist, access to advanced PD treatments is not guaranteed (16). Many general neurologists lack experience in movement disorders and may not always recognize when patients should be evaluated for advanced therapies like deep brain stimulation or infusion therapies. This can result in delays that prevent patients from receiving optimal benefit, as some advanced treatments are most effective when introduced earlier in the disease course (17, 18).

Fortunately, the growing availability of biomarkers and objective diagnostic tools holds promise for improving early and accurate PD diagnosis (19–21). While advances in alpha-synuclein seed amplification assays are in their infancy with limited availability clinically, they may make it easier to diagnose PD earlier supporting PCPs and general neurologists in making the diagnosis. However, their increasing availability will not eliminate the need for timely specialist input as they will likely complement clinical diagnostics rather than replace them. Rather, as biomarker testing does become more available, there will be a greater need for structured referral pathways and enhanced care models to ensure that patients receive diagnostic confirmation, and appropriate management at the primary care and general neurology levels in an equitable way, while also facilitating timely access to MDS when necessary.

## The specialist shortage

The neurology workforce faces significant challenges (22). As of 2012, there were approximately 16,366 neurologists in the United States, with projections indicating a 19% shortfall by 2025 (22, 23). According to a 2013 American Academy of Neurology Workforce Survey, only six states had a neurologist supply that met or exceeded the estimated demand, while 31 states (62%) required at least 20% more neurologists than were available. By 2025, projections indicated that 41 states would face a neurologist shortage, with 88% of these states experiencing a mismatch exceeding 20% (22, 23). This problem is not unique to the United States. In Asia, despite housing 60% of the world’s population, the continent has only 20% of the world’s neurologists, with countries like Bangladesh, Cambodia, and India having fewer than one neurologist per million people (24, 25). European countries face similar challenges as well. England has one neurologist per 50,000 people, whereas France and Germany have one per 25,000 (24). Given that MDS represent only a small subset of neurologists, the capacity to provide specialized care for PD and related conditions is likely even more limited.

Geography plays a significant role in access to movement disorder care. Specialists are heavily concentrated in urban medical centers, leaving many rural and underserved areas without sufficient neurologist coverage. One million Americans have a diagnosis of PD, yet a 2023 study found that there are only 660 movement disorders specialists practicing in the U.S., with only six serving rural areas (7). Patients in rural areas are 40% less likely to receive care from a MDS than their urban counterparts (26). As an example, at institutions such as the University of Kentucky, patients travel an average of 115.5 miles to the Movement Disorders Clinic, with some traveling over 600 miles for this type of specialist care (6).

## Insurance and financial barriers

Even when movement disorder specialists are available, financial and insurance-related barriers further limit access, particularly for marginalized and high-risk groups (27). Many PD patients in the US rely on Medicare, Medicaid, or safety-net hospitals, which often have long wait times for neurology referrals. Additionally, out-of-pocket costs for specialist visits, advanced therapies, and transportation create further burdens for low-income patients (27–29). These burdens disproportionately affect patients from historically underserved communities, compounding existing health disparities (8, 30–32). Individuals with lower income, limited education, or unstable employment are less likely to have continuous coverage or the means to absorb unexpected medical expenses (33, 34). These patients may also face competing priorities, such as caregiving, housing insecurity, or inflexible work schedules, that further reduce their ability to access specialty care (33). Socioeconomic status, geography, and limited health literacy often intersect to create structural inequities in accessing timely, expert PD care (8, 35–37).

## Differences in referral patterns

Differences in referral patterns across patient populations are increasingly recognized as a key contributor to disparities in healthcare access. These differences likely stem from a complex interplay of individual provider behaviors, institutional practices, and broader systemic structures. In a cross-sectional study of Medicare beneficiaries, PCPs shared Black patients with fewer specialists relative to White patients (38). In PD specifically, duration from symptoms’ onset to MDS visit for women was 61% greater than for men reflecting referral delays (39). There are known, and persistent, disparities in the rate of deep brain stimulation surgeries between racial groups (10, 31, 40). Since deep brain stimulation is typically offered primarily through movement disorders centers, referral delays and biases likely play at least a part in these inequities. Understanding how referral decisions are made, and how they vary based on clinician, patient, and system-level factors is critical to addressing inequities in PD care.

## Potential solutions to ensure equitable access to MDS

Ensuring timely and equitable access MDS requires a multifaceted approach that addresses both structural limitations and clinical practice gaps. While increasing the MDS workforce is a long-term goal, near-term solutions must focus on optimizing existing resources, improving referral efficiency, and supporting PCPs who are often the first and only point of contact for many PD patients. The following strategies offer actionable pathways to extend specialist expertise, streamline care delivery, and reduce access barriers across diverse clinical and community settings.

### Standardized referral pathways

Creating clear, evidence-based referral criteria can help guide PCPs and general neurologists in identifying when to refer patients to an MDS. Clinical decision support tools have been shown to be effective in guiding referrals for subspecialty care (41). Integrating clinical decision-support tools into electronic health records prompting PCPs to consider referrals based on disease progression and symptom complexity (medication dosing, number of medications tried, years since diagnosis) is needed. The “5-2-1” criteria for advanced therapies offer a practical framework for identifying patients who may benefit from referral to an MDS (42–44). These criteria include: taking more than five doses of levodopa per day, experiencing more than 2 h of “off” time per day, or having more than 1 h of troublesome dyskinesia per day. Algorithms to identify these criteria can be built into EHR platforms, triggering alerts to consider referrals when the criteria are met.

As part of this standardization, it is important to also consider stepped care models that integrate into the workflow of prompted referrals. This model has been explored in “fast track” clinics for advanced therapies such as deep brain stimulation. In systems where MDS access is delayed or restricted, establishing structured, stepwise referral protocols becomes critical. These should include guidelines on timing (e.g., referral within 6–12 months of diagnosis, or at signs of symptom progression) and content (e.g., standardized symptom checklists or PD severity scores). There are no consensus driven criteria driving such guidelines and these may be difficulty to establish given the heterogeneity of PD. However, specific milestones may be considered when designing such models (Table 1).

**Table 1:** Hypothetical criteria that could be incorporated into workflows that direct referrals.

Potential criteria prompting referral from PCP	Referral outcome
Newly diagnosed	MDS—research screening
Time based—annual evaluations, scheduled (e.g., at time of diagnosis, 1 yr., 3 yr., 5 yr)	General neuro or MDS
Disease metric based—motor symptom progression, nonmotor burden, years of disease, development of fluctuations	General neuro or MDS especially for advanced therapies
Medication based—complexity of medication regimen, number of daily doses	General neuro or MDS especially for advanced therapies
Combination, e.g., “5-2-1” criteria	MDS—advanced therapies

Symptoms of atypical parkinsonisms or other clinical markers that may necessitate sooner MDS input should be factors that can be integrated in these pathways. It should be noted that earlier evaluation has been necessary to allow for recruitment for early disease modifying studies. However, it may be possible to separate research trial screenings from clinical workflows such that being a patient of a MDS is not gatekeeping access to clinical trials.

### Leveraging telemedicine and E-consults

Telemedicine and e-consults provide opportunities to expand access to MDS without requiring in-person visits. These approaches have already been shown to reduce unnecessary referrals and reduce time to specialists’ appointments (45). Telehealth for PD has been shown to be successful in pilot studies from patient and clinical care perspectives (46).

Expanding e-consult programs and adapting them to better serve patients with neurodegenerative diseases is needed. E-consults allow PCPs to receive asynchronous specialist input to guide management. This platform also presents the opportunity to facilitate education regarding management in the form of small, minimally burdensome clinical pearls. Studies have also shown that even though e-consults are patient specific consultations, there is longitudinal learning integrated into the process (47). Integrating clinical decision-support tools into electronic health records can standardize referral criteria, prompting providers to refer patients based on disease progression markers such as motor fluctuations, medication adjustments, or non-motor symptoms (41). There are mechanisms for billing with e-consults and telehealth that need to be advocated for to allow these services. These mechanisms can be adapted to minimize the burden of “unpaid work” on clinicians while still allowing access to specialist care.

An important complement to telemedicine and e-consults is the use of objective PD monitoring tools. Numerous commercially available technologies, such as wearable sensors, smartphone-based assessments, and home monitoring systems, can provide continuous or episodic data on motor symptoms, fluctuations, and medication response (48–51). These tools offer a more representative and granular picture of a patient’s condition, particularly when an MDS has not evaluated the patient in person.

### Addressing financial and system-level barriers

To ensure these solutions are truly accessible, parallel efforts must address the financial and systemic constraints that limit care for low-income and marginalized populations (52). Medicaid reimbursement policies should be expanded to cover telehealth and e-consult services for PD care (22, 53). Transportation assistance programs can reduce geographic barriers (54, 55). Simplifying prior authorization processes and improving transparency around coverage could help reduce delays in care, particularly for patients with limited health literacy, who may struggle to navigate complex insurance requirements. These efforts must be paired with broader policy reforms that align reimbursement with patient-centered, accessible models of neurological care.

### Community engagement and education

Education is a key component of improving PD care and MDS access. Increasing awareness of PD symptoms, treatment options, and referral pathways can help reduce delays in diagnosis and specialist evaluation. This education needs to be at the provider, community, and patient levels.

Expanding provider education through initiatives like the Extension for Community Healthcare Outcomes program can enhance diagnostic accuracy and timely referrals (56). Incorporating education into eConsults presents an opportunity to increase physicians’ knowledge about PD.

Patients and caregivers play a critical role in recognizing disease progression and advocating for advanced care. Community outreach—through support groups, webinars, and digital platforms—can empower patients with the knowledge needed to seek specialized care. Patient-facing decision aids should be widely available to help individuals identify when specialist input is necessary. These tools, accessible via patient portals or advocacy groups, can streamline the referral process and encourage timely intervention.

Health literacy and access to information vary widely, with underserved populations facing the greatest barriers to specialist care (10, 12, 14, 57). Culturally tailored educational programs, translation services, and partnerships with community health organizations can bridge these gaps. Training community health workers to recognize PD symptoms and guide patients through the referral process can further improve access in rural and underserved areas.

## Conclusion—call to action

**Figure 1:**
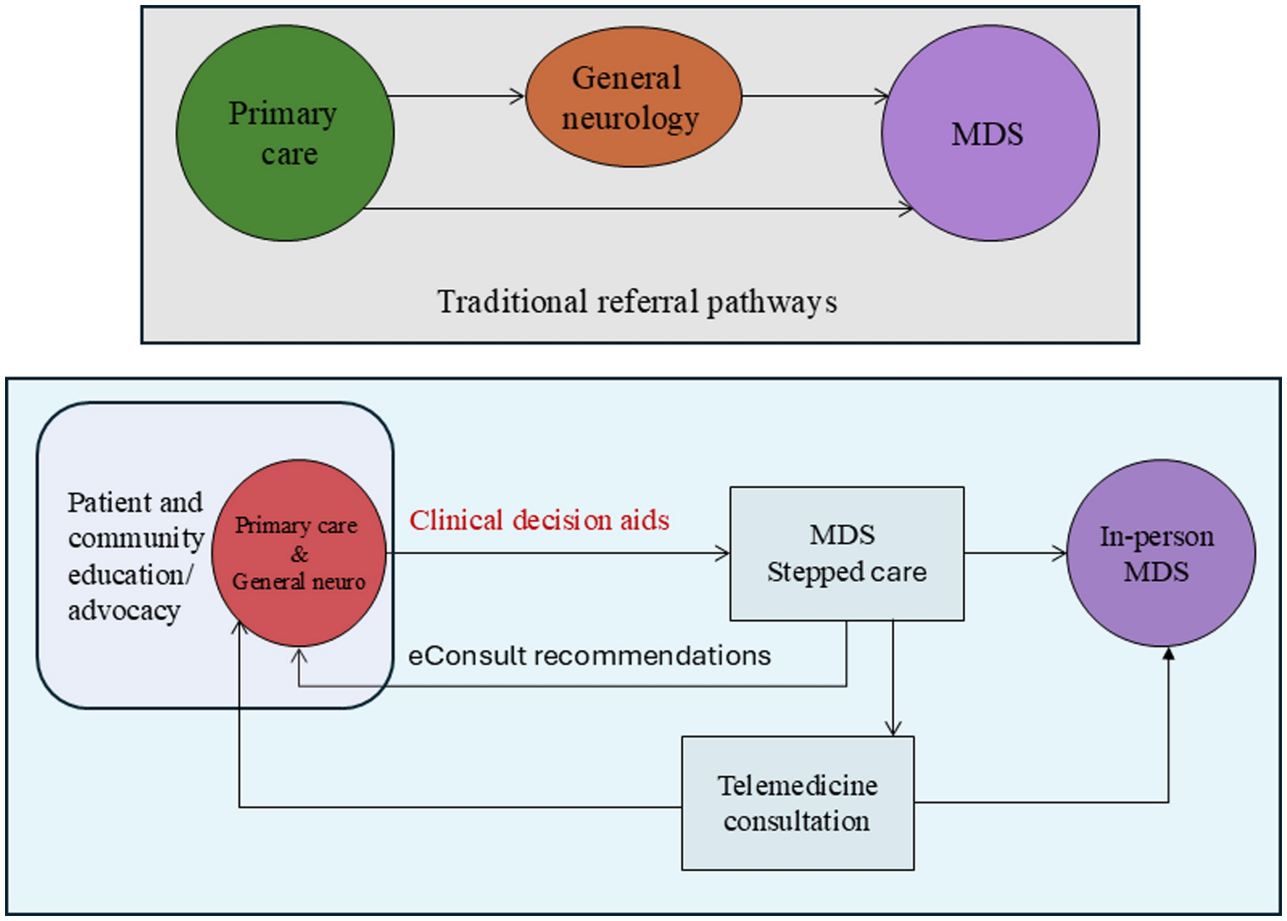
Traditional referral pathways for in-person consultations (top). Structured, stepped care model of subspecialty referral pathway (bottom). MDS, movement disorders specialist.

Ensuring equitable access to specialist care requires multilevel changes that need to take into account an evolving healthcare landscape and disease burden. Standardized referral pathways, expanded telemedicine, e-consult integrations, insurance reforms, and enhanced provider and patient education are critical steps toward improving care outcomes. Solutions include developing and testing novel models of stepped care (Figure 1).

Healthcare providers must integrate decision-support tools and telehealth solutions into routine practice. Policymakers and institutions should prioritize reimbursement for these services as well as funding for specialist training and outreach initiatives. Part of the priority needs to include diversifying the workforce as well. Patients and caregivers must be empowered with the knowledge to self-advocate for timely and appropriate care.

Collaboration among healthcare professionals, advocacy organizations, and policymakers is essential to dismantling barriers to specialist care. By acting now, we can build a more accessible, equitable, and effective care system for all individuals living with not just PD, but other neurodegenerative diseases as well.

## Data Availability

The original contributions presented in the study are included in the article/supplementary material, further inquiries can be directed to the corresponding author/s.
